# The Pros and Cons of Online Competitive Gaming: An Evidence-Based Approach to Assessing Young Players' Well-Being

**DOI:** 10.3389/fpsyg.2021.651530

**Published:** 2021-05-10

**Authors:** Sarah Kelly, Thomas Magor, Annemarie Wright

**Affiliations:** ^1^University of Queensland Business School, Brisbane, QLD, Australia; ^2^Honorary Fellow, Melbourne School of Population and Global Health, The University of Melbourne, Melbourne, VIC, Australia; ^3^Victorian Health Promotion Foundation, Melbourne, VIC, Australia

**Keywords:** eSports, internet gaming, health, well-being, adolescents, youth

## Abstract

This research addresses a lack of evidence on the positive and negative health outcomes of competitive online gaming and esports, particularly among young people and adolescents. Well-being outcomes, along with mitigation strategies were measured through a cross sectional survey of Australian gamers and non-gamers aged between 12 and 24 years, and parents of the 12–17-year-olds surveyed. Adverse health consequences were associated with heavy gaming, more so than light/casual gaming, suggesting that interventions that target moderated engagement could be effective. It provides timely insights in an online gaming landscape that has rapidly evolved over the past decade, and particularly during the COVID-19 pandemic, to include the hyper-connected, highly commercialized and rapidly growing online gaming and esports sector.

## The Pros and Cons of Online Competitive Gaming: An Evidence-Based Approach to Assessing Young Players' Well-Being

Esports as an increasingly popular form of competitive online gaming and can be defined as “… a form of sports where the primary aspects of the sport are facilitated by electronic systems; the input of players and teams as well as the output of the esports system are mediated by human-computer interfaces.” (Hamari and Sjöblom, 2017, p. 211). Its commercialization, globalization and popularity has grown exponentially, evolving to become both a leisure and professional activity (Reitman et al., 2020). Seo et al. (2019) assert that as computer game consumption becomes more complex, interactive and ubiquitous within a context of online and mobile gaming, research focussed upon interaction among consumers, technology, culture, and well-being is critical. Examination into this gaming consumption and its well-being outcomes extend to whether gaming may enhance well-being (e.g., Howes et al., 2017), and on the other hand, manifest as addictive consumption (Frölich et al., 2016). Compounding potential issues of addiction, online gaming is also associated with sedentary lifestyles, poor sleep, unhealthy dietary habits and is a known risk factor for obesity (Boyle et al., 2016; Hoyt et al., 2018; Taylor, 2018; Jeong et al., 2019).

Contrary to this view, some research suggests that competitive gaming can have positive outcomes upon players, relying on heavy exertion, cognitive spatial awareness, decision-making under pressure, and is, by definition, a collaborative endeavor [for a review, see Boyle et al. (2011) and Halbrook et al. (2019)]. Video game playing is associated with stronger cognitive abilities and certain positive neurological effects (Nuyens et al., 2019) and when gaming is balanced with physical activity (Halbrook et al., 2019). Social factors associated with video gaming, based mainly on samples of young people with internet gaming disorders (or high gaming frequency), include poorer social skills, lower educational attainment, behavioral problems, and fewer friends offline, but can also include increased social networks from online friends (e.g., Lobel et al., 2017; Van Den Eijnden et al., 2018).

Psychological and physiological effects of participation in online gaming have attracted the attention of policy makers, health practitioners, and the community alike, fuelling a need to provide evidence-based research. A lack of governance in esports potentially compounds well-being issues associated with gaming. Unlike traditional sports, control of the content and accessibility of gaming titles rests largely with game publishers (Hollist, 2015; Hall Wilcox, 2017). This research is particularly important during the current COVID-19 pandemic, which has seen an escalation in online gaming participation due to isolation restrictions (Forrester, 2020). While past research has focussed upon physical health outcomes associated with video gaming among adolescents and young adults, none has examined outcomes associated with different forms of gaming participation in the new gaming era, and their potential differential health outcomes across different consumer segments, including heavy users, casual users and non-gamers.

The aim of this research is to therefore address a deficit of empirical knowledge on the well-being outcomes associated with gaming competitively, whether recreationally or intensively, among young people. Unlike traditional sports, in which athletes who spend long hours training are praised for their dedication, spending an excessive amount of time gaming could be considered unhealthy (American Psychiatric Association, 2013; World Health Organisation, 2018). The weight of research however, suggests an optimal well-being gamer profile reflecting more recreational, or casual engagement, in contrast to heavy engagement with gaming (e.g., Longman et al., 2009; Halbrook et al., 2019). Moreover, it is possible that this optimal gamer profile may actually lead to better well-being outcomes relative to non-gamers. We therefore test the following hypotheses in the context of competitive online gaming in our study:

H1: That casual gamers will exhibit less harmful well-being outcomes than heavy gamers.
H2: That non-gamers will exhibit less harmful well-being outcomes than heavy gamers.
H3: That casual gamers will exhibit less harmful well-being outcomes than non-gamers.

To test these hypotheses, we undertook a cross-sectional online survey of young Australian gamers and non-gamers aged between 12 and 24 years and parents of minor participants.

## Method

### Participants and Procedure

An online survey was conducted *via* opt-in “research only” online panels. The in-scope population for the survey was residents of the state of Victoria, Australia who are parents of 12–17 year olds, their children aged 12–17 years old (hereby referred to as minors), and young adults aged 18–24 years old. Panelists were recruited via a blend of print media, online marketing initiatives, direct mail, social media platforms, affiliate partnerships, personal invitations, and a range of other *ad-hoc* initiatives. Respondents received a nominal incentive for their participation in line with panel guidelines. The survey was conducted from 31 May 2019 to 11 June 2019 and received university ethical clearance. Responses from the parent and minors were compared for consistency, however, we mostly report on competitive online gaming outcomes based on responses collected directly from the youth gamer's themselves. The final sample included 905 respondents comprising parents of minors (*n* = 316, 65.2% female), minors aged 12–17 years (*n* = 184, 37.5% female), and young adults aged 18–24 years (*n* = 405, 69.6% female).

### Measures

The survey captured the extent of gaming behaviors, the contexts in which online competitive games are played, attitudes toward online competitive gaming, general health/lifestyle measures, and demographic information (see Table 1 for a summary of measures). The survey took ~10 min to complete. The number of panelists who responded to the survey invitation as a proportion of total invitations was 12.4%, which is an acceptable rate for online surveys conducted *via* non-probability panels (Pennay et al., 2018).

**Table 1:** Description of key survey measures.

**Variable**	**Source**
Respondents' general life satisfaction using	The 11-point, single item scale ranging from 0 = “completely dissatisfied” 10 = “completely satisfied” in reponse to the statement, “thinking about your own life and your personal circumstances, how satisfied are you with your life as a whole?” originally developed by Diener et al. (1985), well-validated (Lucas and Donnellan, 2012; Jovanović, 2016). Using the reported population average from the VicHealth Indicators Survey (Victoria State Government, 2015, p. 29) for adults (M = 7.80), responses above 7 on the scale of life satisfaction were coded as “high,” and those below the state average were coded as “low.”
Social connection with others	Levels of agreement with the statement “I feel connected with others” on a 6-point Likert scale ranging from 1= “strongly disagree” to 6= “strongly agree” (Nicholson and O'Halloran, 2019). In the version of the survey completed by children, a pictorial representation of social connection using overlapping circles was used.
Medical consultation linked to gaming	Respondents indicated whether they had ever (within their lifetime) visited a health expert in relation to a health condition they considered to be linked to their gaming (including any visits to a physiotherapist, optometrist, psychologist, or general practice doctor in relation to a physical or mental health issue).
Physical Activity	• The number of days in a typical week that rigorous activity was engaged in for at least 1 h derived from a single item physical activity measure (O'Halloran et al., 2020). • Types of physical activities with which respondents engaged • 3. The number of hours spent sitting down on a typical weekday/weekend
Soft drink consumption	Ordinal variable ranging from none to more than five cups per day.
Alcohol Consumption	• Consumed an alcoholic drink in the 12 months prior • Frequency of partaking in heavy drinking (five or more standard drinks in a single session) which was rated on a 5-point scale ranging from never to daily. • Occurrence of underage drinking based on the responses given by both the parents reporting on their child's drinking habits and children reporting about their own drinking habits.
Incidence of smoking	Frequency of smoking was rated on a 5-point scale ranging from never to daily.
The incidence of experiencing trouble sleeping	Recorded on a 5-point ordinal scale ranging from never to three to four times per week.
Bullying as a result of gaming	Respondents were asked whether they had experienced any bullying that occurred at any point in time either online (e.g., on social media, during gaming sessions, etc.) or in person at school or at work. Examples of bullying were provided in the survey. The extent of bullying was dichotomised into high/low levels for those who had experienced bullying, with low frequency defined as “less than once or twice” and high frequency as “more than every few weeks” within the 4 weeks prior to completing the survey.
Mitigation strategies	A simple binary response to whether each of a list of mitigation strategies were being used (parents) (e.g., the setting of time limits on their child's gaming, ensuring physical activity, encouraging open lines of communication).
Gaming addiction	Gaming and Addiction Scale (adapted) for Adolescents (Lemmens et al., 2009). The responses were recorded on the scale from 1= never to 5 = very often with respect to the incidence of various behaviors associated with gaming addiction. The scale items used were are listed below. • Have you ever thought about playing a game for most of the day? • You spend increasing amounts of time on games? • Sometimes, you play games to forget about real life? • Others have unsuccessfully tried to reduce your game use? • You felt bad when you were unable to play? • Have you ever had fights with others (e.g., family, friends) over your time spent on games? • Have you ever found yourself anticipating when you would go online again? • Have you chosen to spend more time gaming instead spending time with others in person? • Have you grades or schoolwork suffer because of the amount of time you spent gaming? [Minors only] • Has your work suffered because of the amount of time you spent gaming? [Young Adults only] • Has your study suffered because of the amount of time you spent gaming? [Young Adults only]

### Data Analysis

Respondents were first grouped based on a screening question “[Has your child/Have you] played an online multiplayer game in the last 3 months?”. Those who answered “yes” were coded as “gamers” (minors *n* = 150, young adults = 250), and those who answered “no” as “non-gamers” (minors *n* = 68, young adults = 155). Only gamers were asked gaming-related questions, while all participants were asked questions about their health and personal demographics.

Within the gaming cohort, responses were divided into subgroups based on their reported daily gaming frequency to examine associations between gaming extent and health and well-being. Individuals were classified as light/casual gamers if they reported 1–2 h or less time gaming on a general weekday and as heavy/frequent gamers if they reported more than 2 h, Participants who reported they had not played an online competitive game in the 6 months were classified as non-gamers. Gamers were also asked to indicate at which time of the day they played. For all gamers, the most common times were between 6 and 10 pm. The second most common time of day to play for minors was between 4 and 6 pm, while for young adults was between 10 pm and midnight.

A mixture of primarily cross tabulations using chi-square tests for differences in proportions, *t*-tests and one way analysis of variance (ANOVA) for variables which were measured continuously, were used to determine statistically significant differences in the health and well-being outcomes of the different gamer groups.

## Results

### Gaming Frequency and Differences Between Cohorts

**Figure 1:**
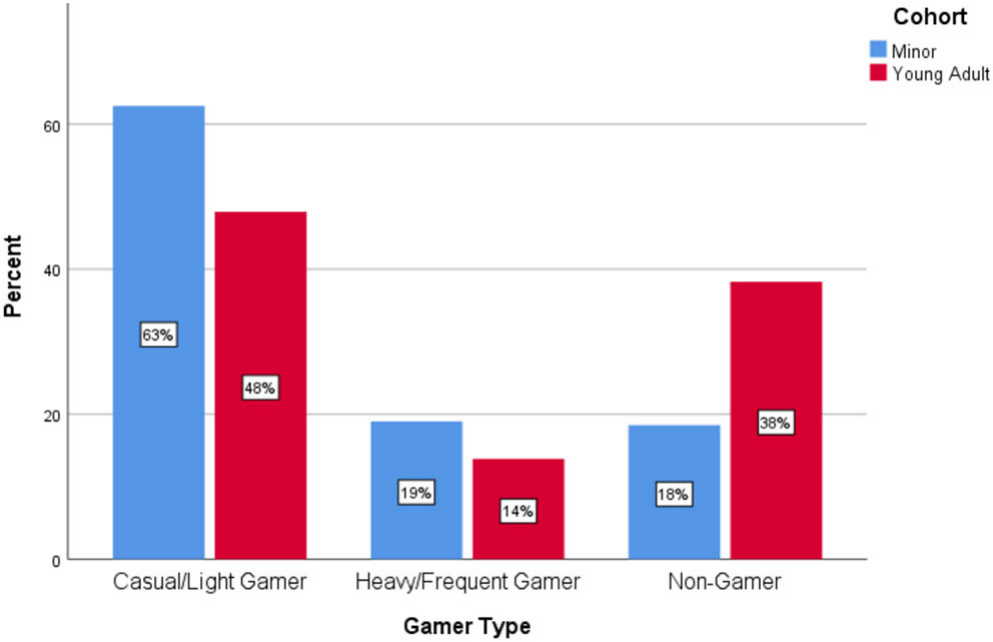
Percentage of gamer types in each cohort.

We conducted quantitative comparisons within each of the cohorts of minors and young adults based on their gaming classification: light/casual gamers, heavy/frequent gamers and non-gamers. Overall life satisfaction, social connection, physical activity, diet, sleep quality, and other activities related to school or work were assessed. In the minor cohort (*n* = 184), 63% (*n* = 115) were light/casual gamers, 19% (*n* = 35) were heavy/frequent gamers and 18% (*n* = 34) were non-gamers. In the young adult cohort (*n* = 405), 48% (*n* = 194) were light/casual gamers, 14% (*n* = 56) were heavy/frequent gamers and 38% (*n* = 155) were non-gamers (see Figure 1).

### Overall Life Satisfaction and Social Connection

The levels overall life satisfaction was first compared between gamer types using an ANOVA for the differences in means between groups. For minors, the results are inconclusive as the assumption of equal variances is not upheld in this cohort as Levene's test indicated unequal variances [*F*_(2,N=177)_ = 5.17, *p* < 0.01], although using Welch's test, which is more robust against unequal means, shows there is an insignificant difference between groups *F*_(2,N=58.2)_ = 1.33, *p* = 0.21.

For young adults, Levene's test shows equal variances *F*_(2,N=400)_ = 0.71, *p* = 0.49, and the ANOVA reveals a significant differences in life satisfaction between gamer types, *F*_(2,N=400)_ = 1.24, *p* < 0.05. Heavy/frequent gamers report the highest level of overall life satisfiaction among young adults scoring an average of 7.46 (*SD* = 2.08) on the 11 point scale, compared to 6.71 (*SD* = 1.83) in casual/light gamers and 6.94 (*SD* = 1.88) in non-gamers.

**Figure 2:**
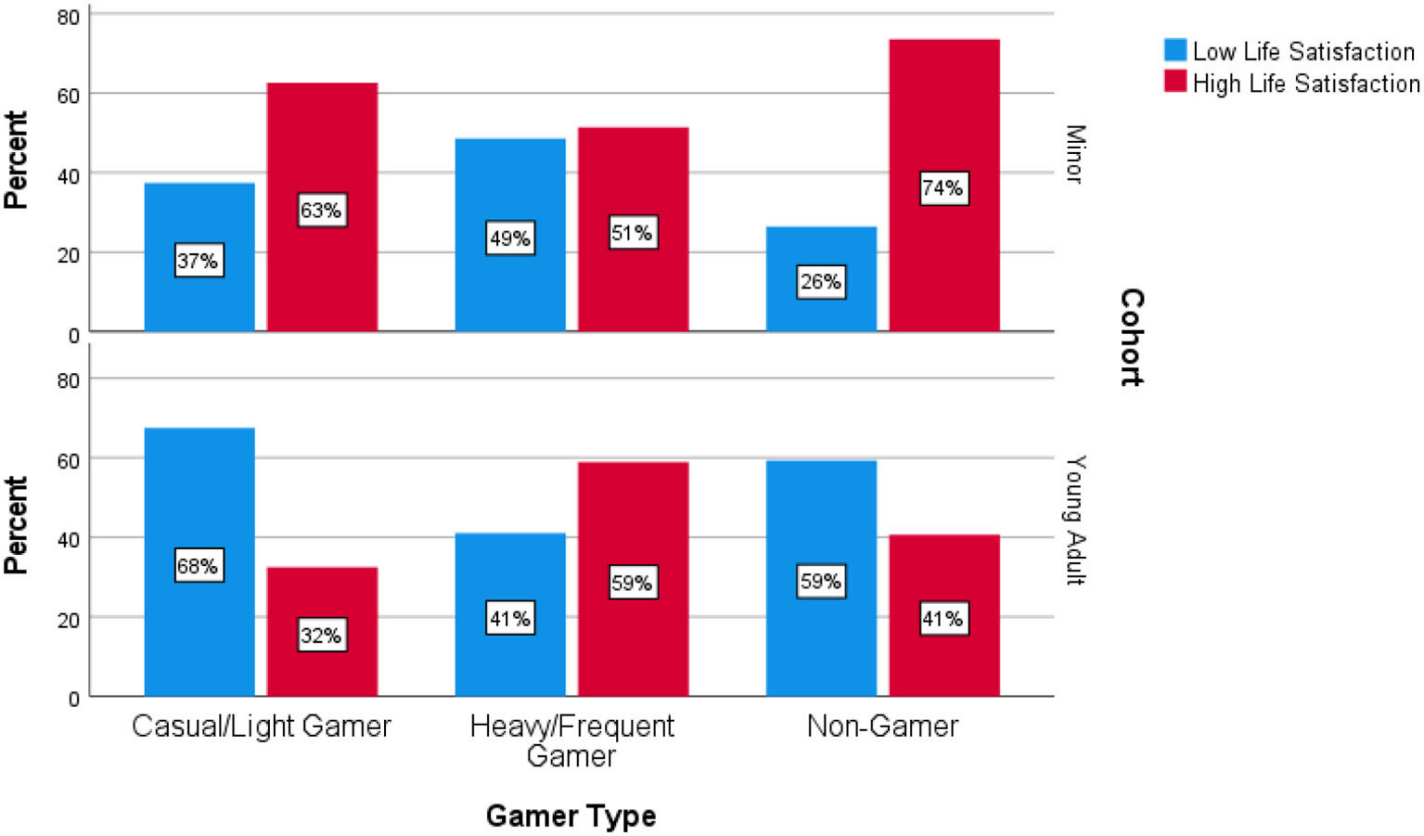
Overall life satisfaction (dichotomised) among minors.

The reported population average from the VicHealth Indicators Survey for adults is 7.80 (Victoria State Government, 2015, p. 29). Using this estimated population average as a basis for dichotomising the responses on overall life satisfaction into “high” and “low” does not lead to a substantially different interpretation of the data for minors, although does it permit for a more robust statistical test as there are above the minimum required cell counts in the contingency table (zero cells have an expected count <5 in both the minor and young adult data). Among minors, the proportions of those reporting a low/high level of life satisfaction did not differ significantly between gaming groups in the minor cohort, ${\text{χ}}_{\left(2,\text{N}=184\right)}^{2}$ = 3.60, *p* = 0.16. For young adults, the interpretation of the data vis-à-vis our interpretation of full scale data does not change. We find heavy/frequent gamers are more likely to be satisfied in life with 59% reporting high life satisfaction compared to 32 and 41% in casual/light gamers and non-gamers respectively with the difference in these proportions being statistically significant, ${\text{χ}}_{\left(2,\text{N}=405\right)}^{2}$ = 0.22, *p* < 0.05, (see Figure 2).

The levels of social connection were compared between gamer types using an ANOVA and *t*-tests for differences in means between groups. No significant differences in the mean levels of social connection between gamer types were found in either cohort. The level of social connection reported by minors averaged 4.73 (*SD* = 0.92) for light/casual gamers, 4.42 (*SD* = 1.37) for heavy/frequent gamers and 4.52 (*SD* = 1.25) for non-gamers on a 6-point scale. The differences in these means were not significantly different, *F*_(2,N=179)_ = 1.26, *p* = 0.29. The mean levels of social connection reported by young adults were 4.34 (*SD* = 1.03) for light/casual gamers, 4.34 (*SD* = 1.39) for heavy/frequent gamers and 4.33 (*SD* = 1.07) for non-gamers on the 6-point scale. The differences in these means were also not significantly different, *F*_(2,N=401)_ = 1.26, *p* = 0.99.

### Physical Activity

Using a one way ANOVA, no significant differences were found within gamer types in either cohort with respect to physically active days. The number of physically active days reported by minors was on average about 3 days, *F*_(2,N=144)_ = 0.76, *p* = 0.47. Physically active days reported by young adults was also on average about 3 days, *F*_(2,N=326)_ = 1.28, *p* = 0.28.

**Figure 3:**
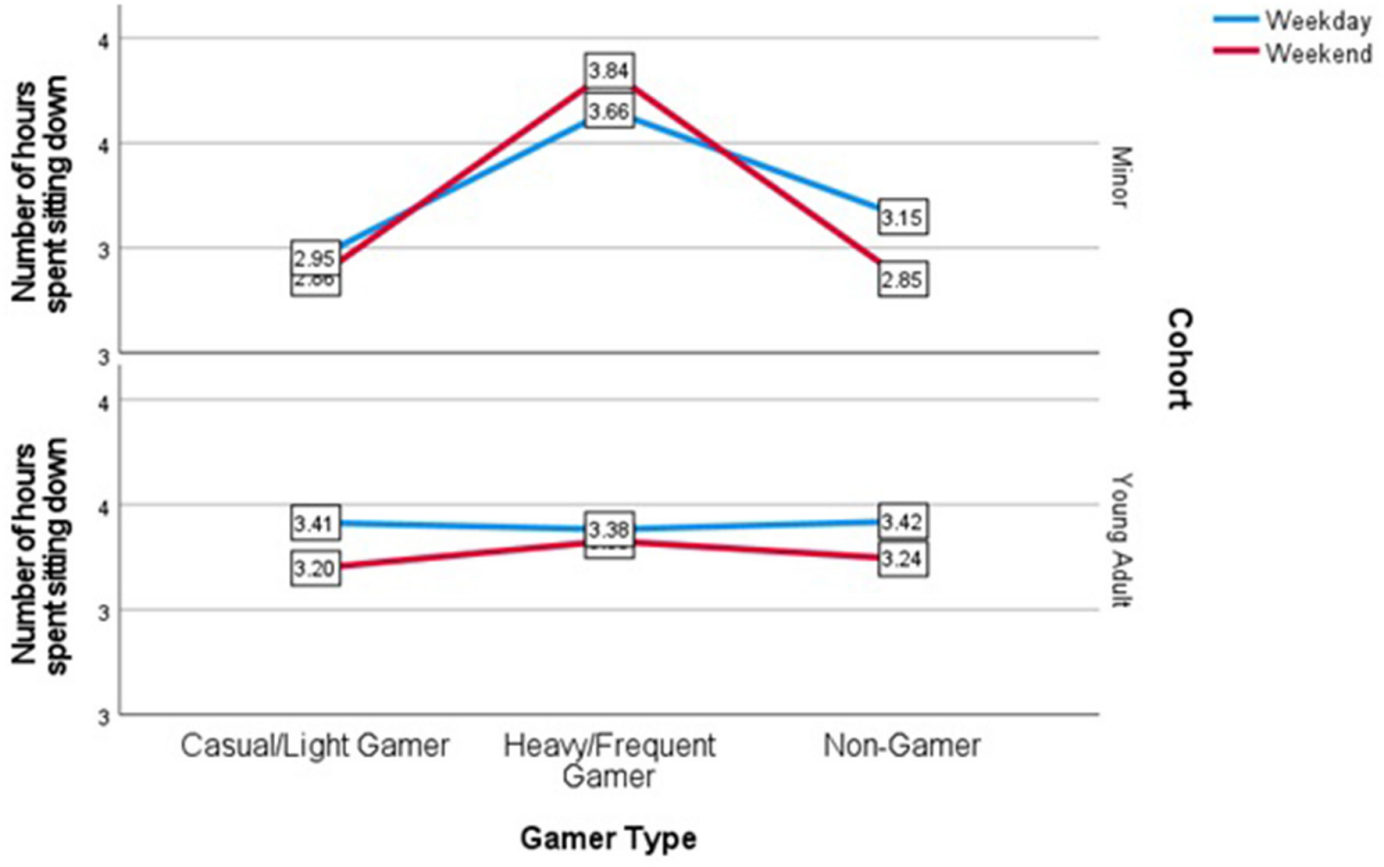
Means plot of hours sitting down on weekdays and weekends.

In relation to minor's inactivity however, significant differences on average hours of sitting down with heavy/frequent gamers sitting on average for 3.84 h (*SD* = 1.88) on weekends, compared to light/casual gamers and non-gamers who reported sitting for 2.95 and 2.85 h (*SD* = 1.62 and *SD* = 1.28) respectively, *F*_(2,N=159)_ = 5.02, *p* < 0.001. There differences in inactivity for minors on weekdays was also significant, but less significant than on weekends, with heavy/frequent gamers sitting on average for 3.66 h (*SD* = 1.04), compared to light/casual gamers and non-gamers who reported sitting for 2.95 and 3.15 h (*SD* = 1.27 and *SD* = 1.26) respectively, *F*_(2,N=159)_ = 5.02, *p* < 0.05. For the young adult cohort, there were no significant differences in the average number of hours sitting on either weekdays or weekends, with *F*_(2,N=395)_ = 0.16, *p* = 0.98 for weekdays, and *F*_(2,N=392)_ = 0.14, *p* = 0.77 for weekends (Figure 3).

### Soft Drink, Alcohol Consumption, and Smoking

**Figure 4:**
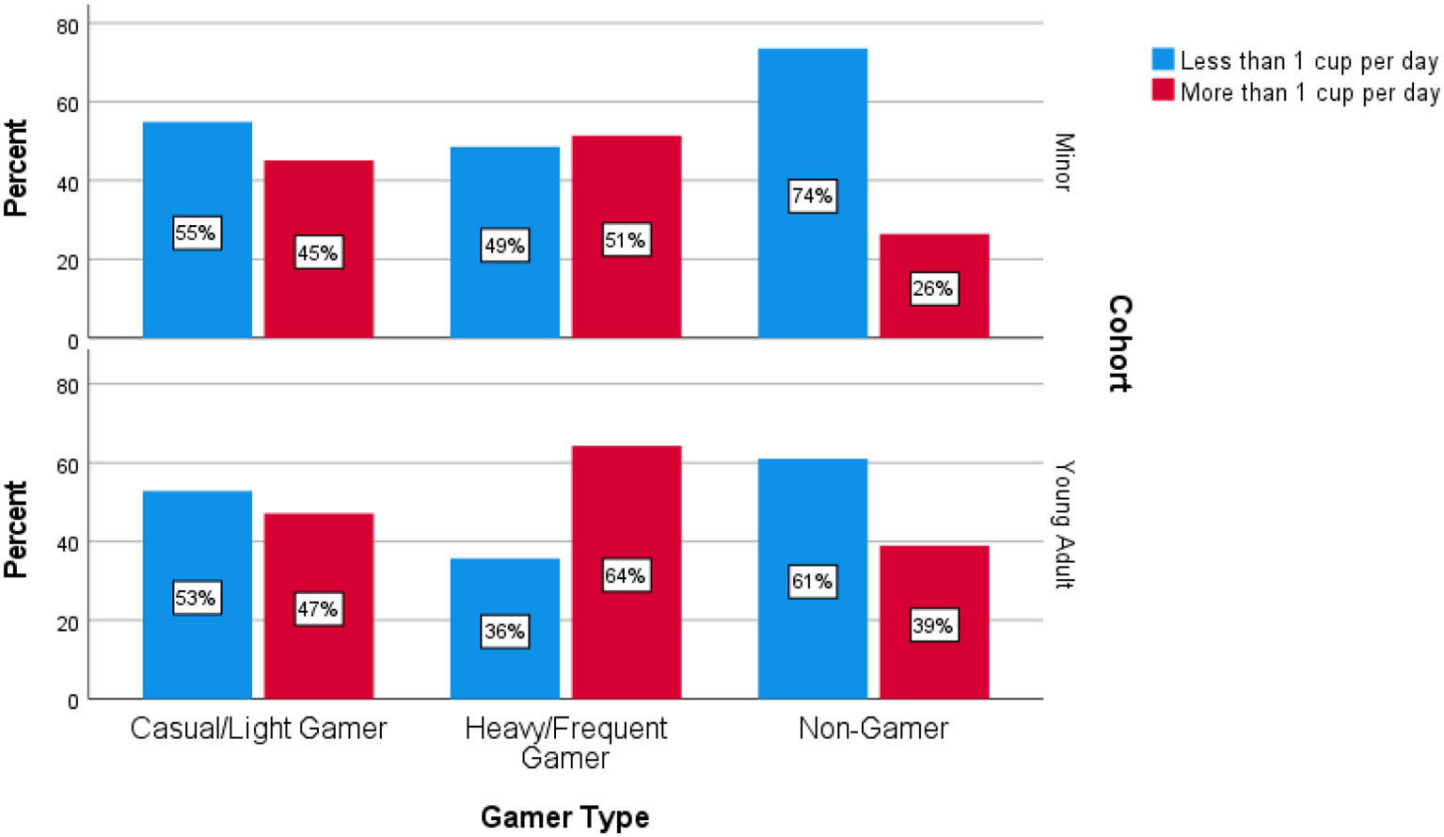
Softdrink consumption among minors and young adults.

In the young adult cohort, heavy/frequent gamers were more likely to be heavy consumers of soft drink compared to both light/casual and non-gamers with 64% of heavy/frequent gamers reporting drinking more than 1 cup of softdrink per day, compared to 47 and 39% in the casual/light and non-gamer cohorts respectively. The differences in these proportions amongst minors was approaches statistical significance with ${\text{χ}}_{\left(2,\text{N}=182\right)}^{2}$ = 5.02, *p* = 0.08. This is driven by the high percentage (74%) of non-gamers who report drinking <1 cup of softdrink per day, however the difference between the two gaming groups is minimal, as summarized in Figure 4.

Among young adults, heavy/frequent gamers reported a significantly higher frequency of engaging in harmful drinking, with 60% reporting drinking more than five standard drinks on a more than monthly basis compared to 50 and 40% in the casual/light and non-gamer groups respectively. The differences in these proportions was statistically significant, ${\text{χ}}_{\left(2,345\right)}^{2}$ = 6.45, *p* < 0.05. For minors, the incidence of alcohol consumption was too low to conduct any robust statistical tests between gaming groups, so we conclude it unlikely there is an association between gaming and alcohol consumption in minors. We checked for consistency between minor's reporting and their parents reporting with respect to own their children's smoking habits and alcohol consumption. We must consider that these measures are self-reported which may impact the accuracy of this measurement.

From our sample 10 minors reported having used a tobacco product at least once, with 14% heavy/frequent gamers reporting having smoked compared to only 4% of casual/light gamers and no non-gamers reported smoking. As the difference in these proportions is statistically significant, ${\text{χ}}_{\left(2,\text{N}=183\right)}^{2}$ = 7.49, *p* < 0.05, further research into the incidence of smoking among minors who are heavy/frequent gamers. Our data further suggests the incidence of smoking increases into adulthood as 48% of heavy/frequent gamers report having smoked tobacco compared to 28% in casual/light gamers and 22% in non-gamers, ${\text{χ}}_{\left(2,\text{N}=403\right)}^{2}$ = 13.7, *p* < 0.05.

### Sleep Quality

The levels of sleep was compared between gamer types using an ANOVA for the differences in means between groups. The assumption of homoscedasticity is satisfied for both groups. For minors there are differences in their reported sleep quality dependent on their gaming extent, *F*_(2,N=179)_ = 3.67, *p* < 0.05. Among minors, heavy/frequent gamers report the most trouble sleeping with the mean for heavy/frequent gamers being 3.63 (*SD* = 2.08), which is trouble sleeping on an almost weekly basis. For casual/light gamers the mean value was 2.74 (*SD* = 1.80) and for non-gamers it was 2.52 (*SD* = 1.95), both corresponding to experiencing trouble sleeping about once a month. No significant differences in sleep emerged for the young adults group with the average level reported corresponding to experiencing some trouble sleeping about once every 1–2 weeks for young adults.

### Bullying Related to Online Gaming

The reported occurrence of bullying among minors reveals 16.9% have experienced some form of bullying related to online gaming. This figure is 15.1% among young adults.

The frequency of bullying was compared between gamer types using an ANOVA for the differences in means between groups. The assumption of homoscedasticity is satisfied for both groups. For minors, heavy/frequent gamers reported being bullied on average “every few weeks”, *M* = 3 (*SD* = 1.18) compared to casual/light gamers reporting being bullied closer to only “once or twice”, *M* = 1.90 (*SD* = 0.94). These differences are statistically significant in the cohort of minors, *F*_(2,N=22)_ = 5.71, *p* < 0.05. The frequency of bullying between gamer groups was not significantly different in young adults, *F*_(2,N=53)_ = 0.56, *p* = 0.46. Non-gamers were not asked whether they had experience bullying related to online competitive gaming.

### Parental Mitigation Strategies

Parents were asked about mitigation strategies they might be using to reduce the extent of their child's gaming. The response format was a simple binary response to whether each of a list of mitigation strategies were being used (e.g., the setting of time limits on their child's gaming, ensuring physical activity, encouraging open lines of communication). We conducted paired samples analysis using data from only those parents for whom a corresponding minor could be matched in the data (*n* = 184). Some mitigation strategies used by parents were found to be significantly associated with less harmful reported behaviors by minors. These include parents setting time limits, which was associated with lower reported daily gaming hours of between 1 and 2 h by minors whose parents set limits compared to between 3 and 4 h per day by those whose parents do not, *F*_(1, 132)_ = 10.12, *p* < 0.05. Parents ensuring their children are physically active was associated with minors reporting a greater number of days they are physically active, *F*_(1, 125)_ = 7.92, *p* < 0.05.

### Problematic Gaming

With only a few exceptions within the young adult cohort, heavy/frequent gamers reported significantly higher levels of problematic gaming compared to light/casual gamers. For example, amongst gamers who “[…] thought about playing a game for most of the day,” 24% were heavy/frequent gamers while only 10% were light/casual gamers. This difference in proportion was statistically significant ${\text{χ}}_{\left(1,\text{N}=136\right)}^{2}$ = 17.66, *p* < 0.001. A similar pattern persists through all of the gaming and addiction scale items in the minor cohort. The exceptions within the young adult cohort are the items: “Sometimes, you play games to forget about real life?,” “Others have unsuccessfully tried to reduce your game use?,” “Have you felt bad when you were unable to play?” and “Have you chosen to spend more time gaming instead spending time with others in person?”. For these items, there were no significant differences between gamer types (heavy/frequent vs light/casual gamers) in the young adult cohort.

As a more general measure of health-related impacts of online competitive gaming, we also asked gaming respondents to indicate whether they had ever (within their lifetime) visited a health expert in relation to a health condition they considered to be linked to their gaming. Amongst children, 10 individuals representing 7.5% of the total population of children reported having sought medical advice related to their gaming, 80% of which were heavy/frequent gamers compared to 20% light/casual gamers. Of those who did not indicate ever having sought medical advice related to their gaming, 43.1% were heavy/frequent gamers compared to 56.9% light/casual gamers and the differences in these proportions are statistically significant for children (χ^2^ = 5.07, *df* = 1, *p* < 0.05). In the young adults cohort, 19 individuals representing 8.7% of the total population indicated having sought medical advice for a health concern connected to their gaming, 73.7% of which were heavy/frequent gamers compared to 26.3% of light/casual gamers. Of those who had not sought health advice, the proportions are more evenly distributed with 47.2% of heavy/frequent gamers and 52.8% of light/casual gamers. The differences in these proportions is statistically significant in the young adult cohort (χ^2^ = 4.85, *df* = 1, *p* < 0.05).

## Summary

Based on these results, both hypothesis 1 and 2 were supported. Casual gamers and non-gamers reported less harmful well-being outcomes (e.g., less sedentary time on weekends for minors, lower soft drink and alcohol consumption, lower proportions of smokers, and less reported trouble sleeping) compared to heavy gamers. On the other hand, hypothesis 3 was not supported, as no notable differences on health and well-being outcomes between casual gamers and non-gamers emerged.

## Discussion

The aims of this research were to gain insight into the positive and negative well-being outcomes associated with online competitive gaming among young players, in addition to identifying suitable mitigation strategies. Overall, our findings suggest that gaming engagement in moderation is preferable for health across minor and young adult cohorts. In fact, casual/light gamers reported similar health and well-being outcomes to non-gamers.

There are similarities and differences between the two cohorts (minors and young adults) with respect to the specific associations between heavy gaming and adverse well-being outcomes. In both cohorts, there is a higher likelihood for heavy gamers to be heavy consumers of soft drink, to have smoked at least once and to have seen a health professional for gaming related health problems. Amongst young adults, heavy gamers were more likely to engage in heavy drinking which may be due to their exposure to higher rates of alcohol advertising (Kelly and Van der Leij, 2020). In regard to bullying, the only significant difference was found in the young adult cohort where light gamers were more likely to have experienced bullying than heavy gamers. Another surprising finding was that in the young adult cohort, heavy gamers had significantly higher life satisfaction. Minors who were heavy gamers were more likely to have difficulties sleeping and spend the most amount of time sitting, whilst minors who were light gamers spent the least amount of time sitting (even less than non-gamers). For both cohorts, our findings also reveal that non-gamers were not necessarily more physically active than gamers. This suggests that non-gamers likely spend their sedentary time on other activities.

Results indicate that parental mitigation strategies were effective in relation to determining whether a minor was a casual or heavy gamer. The monitoring strategy of parents setting time limits on their children's gaming frequency is shown to be associated with a reduced number of hours gaming on weekdays according to responses given by both parents and minors, but was not associated with reducing the number of hours minors gamed on weekends. Specifically, minors whose parents impose time limits report closer to 1–2 h of weekday gaming vs. 3–4 h from minors whose parents did not impose limits. In addition, minors whose parents make sure they do physical activity report significantly more engagement in physical activity.

It should be noted that there are some limitations to this study, including the self-reporting and cross-sectional nature of the survey method, requiring a high degree of self-awareness and insight that young people may not necessarily have well-developed. The path from casual gaming to heavy gaming is also not identified due to the cross-sectional design, but would be of interest for future research. Measurement of casual gaming may also underestimate health outcomes associated with gaming, as some of these outcomes are related more generally to screen time, which may not include gaming in isolation. It may therefore be important in future research to consider other screen-based activities in association with online competitive gaming. The small sample size across the cohorts and the sub-categorization into gamer types may have diminished power to conduct robust analyses for some variables. As this research was only intended as an initial snapshot of the current gaming landscape, several items were adapted from more extensive and validated scales due to length restrictions.

Future research is warranted, extending on these findings, to gain causal understanding into relationships identified, such as the role of mitigation strategies, parental monitoring, parental engagement in gaming and the optimal ways to prevent heavy gaming tendencies, whilst not completely restricting gaming engagement. This is particularly important for those young people who have experienced lockdown during the COVID-19 pandemic, when rates of online gaming have increased due to the limited number of social and recreation activities external to home (King et al., 2014; Victoria State Government, 2019). Our findings in relation to competitive online gaming align with earlier research which investigates the protective role of parental media monitoring more broadly (Padilla-Walker et al., 2018). Examination of structural aspects in popular games identified is also needed to gain insight into possible cues driving heavier gaming behavior. As younger cohorts of millennial parents emerge, who are themselves avid gamers, it will be critical to educate them about the influence they may have upon their offspring. Further survey evidence could examine heavy gaming cohorts in a more granular approach, given the negative health outcomes that have been revealed as associated with this cohort. Positive outcomes of casual gaming including what appears to be social connection, self-esteem, and well-being need to be emphasized and examined further, perhaps through observation and in-depth interview studies of gamers, in addition to replication cross-culturally.

Another avenue for future research may concern how those who engage in a casual/light extent of gaming balance other screen and non-screen based activities, compared to those frequent/heavy gamers and those who do not engage with gaming but may engage in other screen based activities (e.g., excessive social media usage). While previous research has found health outcomes associated with gaming behaviors (e.g., Rosen et al., 2014; Boyle et al., 2016), there is limited research which examines lifestyle and well-being associated with gaming, especially in children. In addition, the evolving, highly connected and competitive gaming context associated with esports also warrants examination, along with the significant “passive” engagement through streaming.

## Conclusion

This study provides useful initial insight into the online gaming behaviors and associated well-being outcomes of gaming among minors and young adults. It addresses a gap in knowledge of gaming well-being outcomes in the current age of gaming-mediated entertainment and socialization, and illuminates both behaviors and protective health strategies that ensure positive engagement in gaming. Given the burgeoning participation, commercial growth and professionalization of the gaming industry, this research is timely and relevant in providing empirical evidence of both the positive and adverse health associations with gaming, and typical gaming behaviors. Our results demonstrate differential trends associated with gaming frequency and age cohorts and is among the first to examine the health, lifestyle and social outcomes of online gaming among young gamers. Given the growing participation in playing and streaming competitive online gaming and esports, particularly during the COVID-19 pandemic, further research is needed to monitor gaming behavior and well-being outcomes and build on research such as this to establish causative links between gaming behavior and it's outcomes on health and well-being.

## Data Availability Statement

Raw data will be supplied by the authors upon request.

## Ethics Statement

The studies involving human participants were reviewed and approved by University of Queensland Human Ethics Clearance. Written informed consent to participate in this study was provided by the participants' legal guardian/next of kin.

## Author Contributions

All authors listed have made a substantial, direct and intellectual contribution to the work, and approved it for publication.

## Conflict of Interest

The authors declare that the research was conducted in the absence of any commercial or financial relationships that could be construed as a potential conflict of interest.
